# A Study of the Anatomy of the Eustachian Tube for Its Surgical Approach in Otorhinolaryngology

**DOI:** 10.7759/cureus.38411

**Published:** 2023-05-01

**Authors:** Nahid Yasmin, Monica Baxla, Pankaj K Singh, Rati Tandon, Hare Krishna

**Affiliations:** 1 Anatomy, Mahatma Gandhi Medical College and Hospital, Jaipur, IND; 2 Anatomy, All India Institute of Medical Sciences, New Delhi, IND; 3 Anatomy, All India Institute of Medical Sciences, Jodhpur, IND

**Keywords:** sphenoidal sinus, nasopharynx, inferior nasal concha, atlas vertebrae, eustachian tube

## Abstract

Background: The eustachian tube (ET) is a connection between the nasopharynx and the middle ear behind the inferior nasal concha. It plays an important role in regulating air pressure across the tympanic membrane for proper transmission of sound. The pharyngeal opening of the tube is an important landmark for endoscopic evaluation in patients suffering from chronic otitis media and is also an important anatomical landmark for the transnasal approach to the infratemporal fossa. Hence, the study was done to locate the position of the pharyngeal opening of the ET in relation to various important anatomical landmarks.

Methodology: Hundred (50 right and 50 left sides) adult (60-80 years) formalin-fixed sagittal sections of head and neck specimens were taken for the study, which was obtained during the undergraduate teaching program. The shape, size, and position of the pharyngeal opening of the ET were noted. The distance between the pharyngeal opening of the ET and various anatomical landmarks was measured with the help of the digital Vernier caliper. The mean and standard deviation of all the parameters were calculated and tabulated.

Results: In the present study, a slit-like shape was the most common shape of the pharyngeal opening, present in 62 out of 100 specimens. The difference between the anteroposterior length and vertical height of the two sides showed a statistically significant difference.

Conclusion: The present study will help to locate the position of the pharyngeal opening of the ET during otorhinolaryngological evaluation for performing various surgeries in the middle ear.

## Introduction

The eustachian tube (ET), also known as the auditory tube or the pharyngotympanic tube, is an osseocartilaginous tube connecting the tympanic cavity to the lateral wall of the nasopharynx [1]. The name "ET" was given after an Italian anatomist of the 16th century, Bartolomeo Eustachi, who extended the knowledge of ET, but it was first described by Aristotle [2]. The ET plays a role in the equalization of pressure, oxygenation, and drainage of the tympanic cavity in the middle ear. The ET is derived from the proximal narrow portion of the tubo-tympanic recess, which develops from the first pharyngeal pouch [3]. It is about 36 mm long. One-third of the ET is osseous (12mm) and lies posterolaterally between the anterior tympanic wall and the junction of the squamous and petrous parts of the temporal bone; there it has uneven edges for the attachment of the cartilaginous part [1]. Medially, the bony part is related to the carotid canal [1]. The remaining two-thirds (24 mm) of the tube forms the cartilaginous part, opens into the nasopharynx, and lies anteromedially [2]. The tube runs downward, forward, and medially from the tympanic end, making an angle of 45° with the sagittal plane and 30° with the horizontal plane, which increases with advanced age and the increase in length of the cranial base [1]. The smaller end of the tube is tympanic and is located in the anterior wall of the middle ear, while the pharyngeal end is larger, is slit-like, and is placed in the lateral wall of the nasopharynx around 1.25 cm behind and below the posterior end of the inferior nasal concha.

The ET acts as a pressure equalizer on either side of the tympanic membrane. At rest, the cartilaginous part of the tube remains closed at the nasopharyngeal opening. But during yawning, sneezing, and swallowing, it dilates with contraction of the tensor veli palatine, which can open the ET [4,5].

The epithelium lining of the ET protects the middle ear from infections [6]. ET dysfunction can lead to recurrent infections of the middle ear like otitis media or may cause hearing impairment, especially in children. Adolescents and adults are also affected by ET dysfunction, often due to respiratory tract infections [1]. To treat ET dysfunction, mainly conservative management has traditionally been used. Several other therapeutic procedures have been employed, like laser eustachian tuboplasty and drug application through the transtubal approach [7]. Also, to maintain the patency of the ET catheter, it has been passed either from the tympanic cavity or the nasopharyngeal approach [8].

ET opening (pharyngeal) is an important landmark for endoscopic evaluation in various middle ear and nasopharyngeal diseases and for approaching the infratemporal fossa transnasally. Thus, anatomical knowledge of the ET is important. This present study was undertaken to look for the position of the pharyngeal opening of the ET in relation to various anatomical landmarks in a well-embalmed cadaver.

## Materials and methods

The present study was conducted in the Department of Anatomy at Mahatma Gandhi Medical College & Hospital, Jaipur, and the All India Institute of Medical Sciences, New Delhi, after ethical clearance (ethical clearance no. MGMC&H/IEC/JPR/2022/1141). The study was carried out on 100 formalin-fixed mid-sagittal sections (50 right and 50 left sides) of adult (60 to 80 years old) head and neck specimens.

The ET (pharyngeal opening) was observed for its shape, size (anteroposterior length and vertical height), and position. The pharyngeal opening of the ET is measured with respect to the various anatomical landmarks: distance from the roof of the nasopharynx, sphenoidal sinus, clivus-perpendicular distance, anterior tubercle of the arch of the atlas, the tip of the uvula, and the posterior end of the inferior concha. All the measurements were taken using the digital vernier caliper (DVC). The mean and standard deviation of the ET's distance from various anatomical landmarks were calculated. The collected data were tabulated and analyzed statistically.

## Results

**Figure 1:**
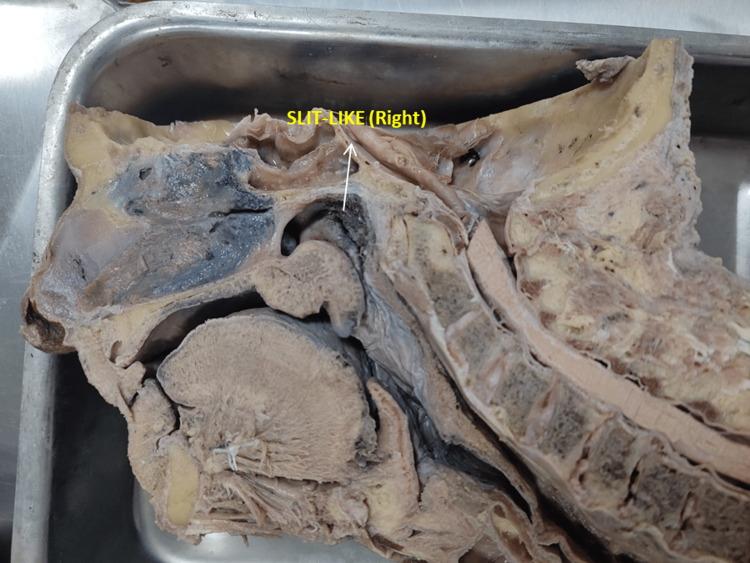
Photograph showing the slit-like-shaped (right) opening of the eustachian tube

**Figure 2:**
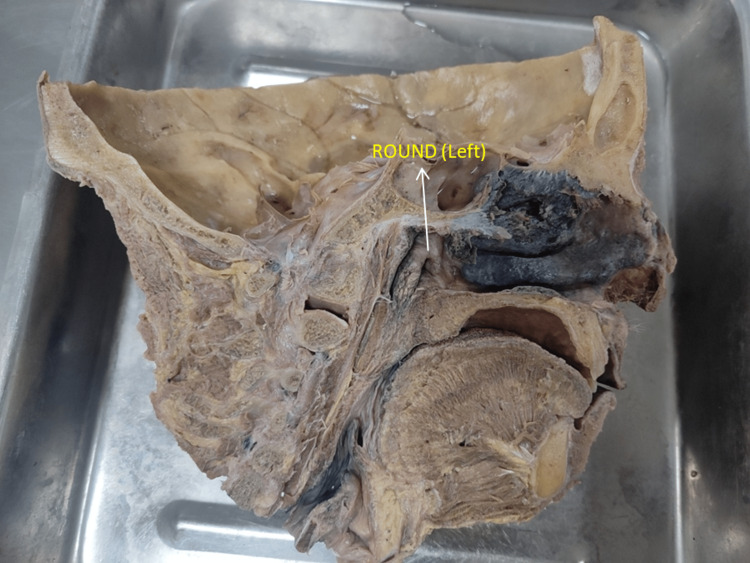
Photograph showing the round-shaped (left) opening of the eustachian tube

**Figure 3:**
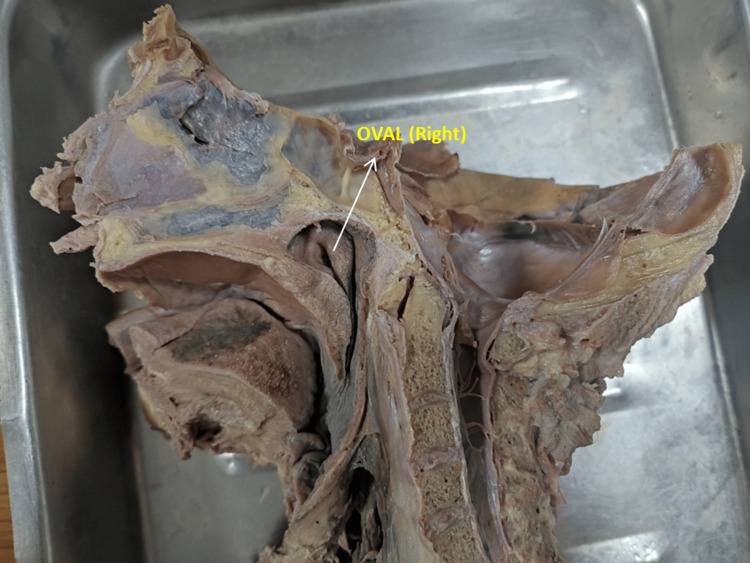
Photograph showing the oval-shaped (right) opening of the eustachian tube

The pharyngeal opening of the ET was studied in 100 formalin-fixed midsagittal sections of the head and neck. The shapes were observed in all the specimens. Various shapes were found, i.e., slit-like, triangular, round, and oval. The commonest was slit-like, and the least common was an oval shape. A slit-like ET was more common on both the right and left sides (Table 1 and Figures 1-3).

**Table 1:** Details of the shape of the pharyngeal opening of the eustachian tube found in the study

The shape of the pharyngeal opening of the eustachian tube	Shapes	Right (n=50)	Left (n=50)
	Slit-like	37	25
	Triangular	05	13
	Round	00	12
	Oval	08	00

The anteroposterior (A-P) length and vertical height were measured. The mean A-P length on the right side was 0.90 and 0.80 cm on the left side, which was statistically significant. The vertical height was higher on the right side (1.10 cm) as compared to the left side (0.90 cm), with a statistically significant difference (Table 2).

**Table 2:** Dimensions of the pharyngeal opening of the eustachian tube

Parameters	A-P length (width, in cm)		Vertical height (cm)	
	Right	Left	Right	Left
Number	50	50	50	50
Minimum value	0.83	0.75	0.9	0.83
Maximum value	0.97	0.85	1.30	0.97
Mean±SD	0.90±0.07	0.80±0.05	1.10±0.20	0.90±0.07
p-value	<0.001		<0.001	

In more than half of the specimens (54), the position of the pharyngeal opening of ET was behind the inferior nasal concha. Out of 100, only 18 were present above the inferior nasal concha (Table 3).

**Table 3:** The position of the pharyngeal opening of the ET with respect to the inferior nasal concha

The position of the pharyngeal opening of the ET	Position	Right (n=50)	Left (n=50)
	Below	10	18
	Behind	32	22
	Above	08	10

**Figure 4:**
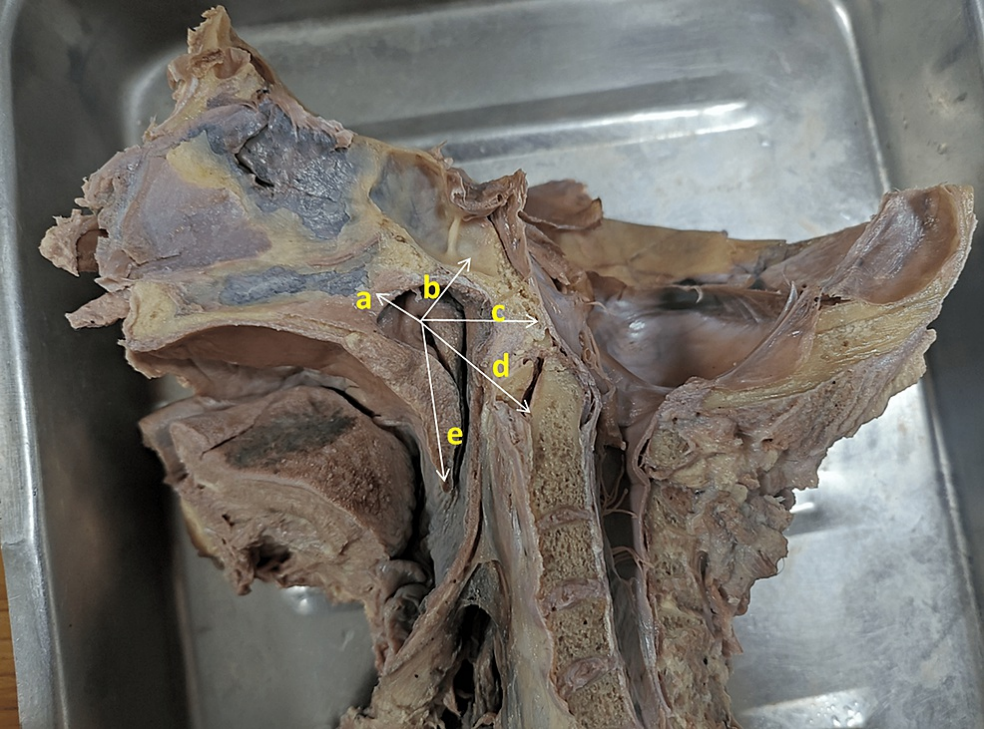
Photograph showing the distance from the center of the eustachian tube to various landmarks (a: roof of the nasopharynx; b: sphenoidal sinus; c: clivus; d: atlas vertebra; e: the tip of the uvula)

**Figure 5:**
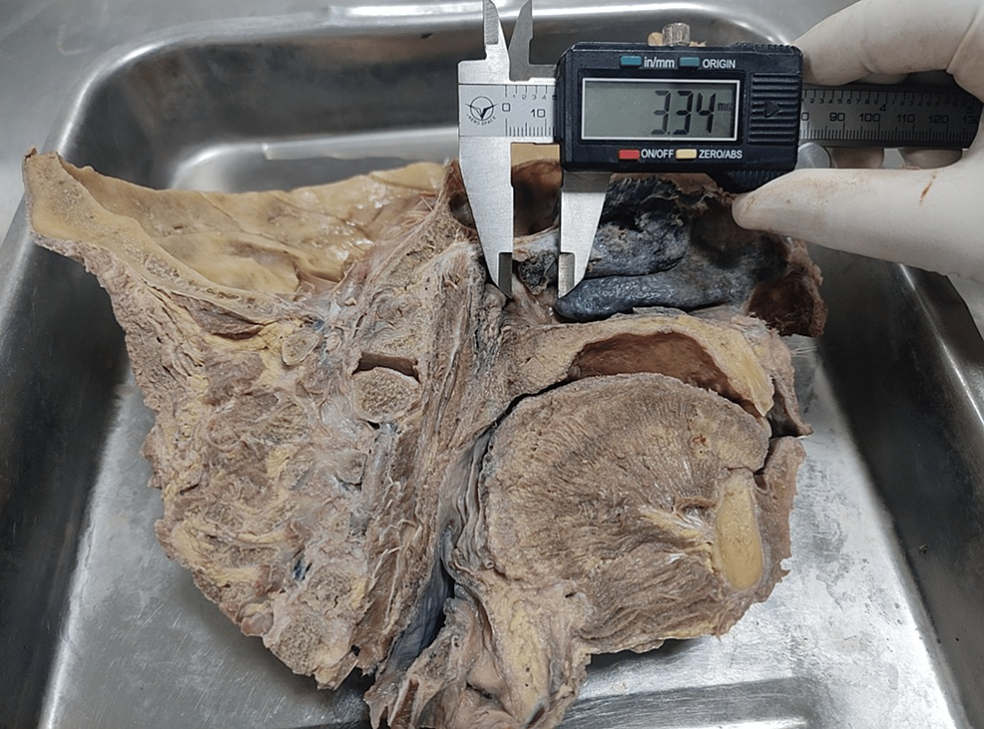
Photograph showing the distance between the ET (in millimeters) and the posterior end of the inferior nasal concha

The location of the pharyngeal opening of the ET was measured from various anatomical landmarks. The mean distance of the pharyngeal opening from the posterior end of the inferior nasal concha was 1.20 cm and 1.30 cm on the right side and left side, respectively, which was statistically significant (Table 4 and Figures 4, 5).

**Table 4:** Morphometric measurements of the pharyngeal opening of the ET

Parameters	From the roof of the nasopharynx (cm)		From the sphenoidal sinus (cm)		From the clivus-perpendicular distance (cm)		From the anterior tubercle of the arch of the atlas (cm)		From the tip of the uvula (cm)		From the posterior end of the inferior nasal concha (cm)	
	Right	Left	Right	Left	Right	Left	Right	Left	Right	Left	Right	Left
Number	50	50	50	50	50	50	50	50	50	50	50	50
Minimum value	1.20	1.10	1.25	1.37	2.33	2.43	2.8	2.9	2.66	2.74	1.13	1.25
Maximum value	1.40	1.30	1.35	1.43	2.47	2.57	3.0	3.1	2.74	2.86	1.27	1.35
Mean ±SD	1.30 ±0.10	1.20 ±0.10	1.30 ±0.05	1.40 ±0.03	2.40 ±0.07	2.50 ±0.07	2.90 ±0.10	3.00 ±0.10	2.70 ±0.04	2.80 ±0.06	1.20 ±0.07	1.30 ±0.05
p-value	0.157		<0.001		<0.001		<0.001		<0.001		<0.001	

## Discussion

Mohite et al. observed that the most common shape of the pharyngeal opening was oval, while the present study reported slit-like as the most common shape [9].

Divya et al. recorded the vertical height to be 0.64 cm and 0.49 cm on the right and left sides, respectively, while the anteroposterior length was 0.31 cm and 0.27 cm on the right and left sides, respectively [10]. In the present study, the vertical height recorded was 1.10 cm and 0.90 cm on the right and left sides, respectively, while the anteroposterior length was 0.90 cm and 0.80 cm on the right and left sides, respectively. These values were statistically significant and higher than the values recorded by Divya et al.

Varalakshmi et al., in 2015, noted that the distance between the posterior ends of the inferior nasal concha was 0.88 cm and 0.84 cm on the right side and left side, respectively, which was slightly less as compared to the present study, i.e., 1.20 cm on the right and 1.30 cm on the left side [11].

Urbantschitsch noted that the distance from the pharyngeal orifice of ET to the anterior nasal spine was 5.3-7.5 cm. He also observed a variation of 0.1 to 1.5 cm in the distance from the posterior end of the inferior turbinate [12].

Various other anatomical landmarks were also measured in the present study; all are slightly in a higher range compared to the previous study by Varalakshmi et al. [11].

Ankolenkar et al. observed the distance of ET with respect to some anatomical landmarks in fetal and adult cadavers. They observed that all the distances measured were higher on the left side except ET to the sphenoidal sinus in fetuses and ET to the tip of the uvula in adults [4]. In our study, all measurements were greater on the left side except for ET to the roof of the nasopharynx.

Alzahrani et al. observed that the average distance from the auditory tube to the uvula center was greater on the left side (23.85 ± 4.05 mm) than on the right side (25.42 ± 6.37 mm). The ET cartilage changed significantly in both shape and size from the young group to the older group [13].

Sadler-Kimes et al. observed that ET cartilage is larger and more elongated in the older group in comparison to being smaller and more oval in the young group. The tensor veli palatine TVP muscle is the primary dilator of ET. In the older group, the TVP muscle superiorly rotated due to aging relative to the ET cartilage. In our study, the most common shape of the cartilaginous opening was slit-like, which may be due to all specimens in our study belonging to the older age group [14].

The junction between the bony and cartilaginous parts of ET (isthmus) is clinically important for surgeons as it serves as an important landmark to avoid carotid artery injury during the surgical intervention of ET, and the junction can be reached by following the lumen of the tube from the nasopharyngeal opening [15]. In patients suffering from chronic otitis media and other cases when ET gets dysfunctional, transnasal endoscopic insertion of a balloon catheter is done to maintain patency [16].

In children, the tube is wider, shorter, and more horizontally placed, causing aspiration of the secretions from the rhinopharynx in mild blockages [17, 18]. In adults, the position and shape are variable.

The fossa of Rosenmüller, a common site of nasopharyngeal carcinoma, lies between the posterior wall of the nasopharynx and the ET [19]. Hence, endoscopic evaluation of this region might be helpful to know about the spread of local tumors or the involvement of surrounding mucosa [4].

Anatomical knowledge of the relationship between ET and various other anatomical landmarks is definitively useful for cranial base surgery and other endoscopic procedures.

## Conclusions

A literature search reveals very little established data on the morphometry of the ET and its clinical relevance. The present study elucidates the importance of the position of the ET and its dimensions, which may be beneficial in otorhinolaryngological evaluation and surgeries. Hence, the result of the present study can be utilized as a reference point to recognize the position of the pharyngeal opening of the ET and its relationship with various anatomical landmarks in endoscopic evaluation.
